# Reciprocal Inhibitory Glomerular Circuits Contribute to Excitation–Inhibition Balance in the Mouse Olfactory Bulb

**DOI:** 10.1523/ENEURO.0048-19.2019

**Published:** 2019-06-11

**Authors:** Zuoyi Shao, Shaolin Liu, Fuwen Zhou, Adam C. Puche, Michael T. Shipley

**Affiliations:** Department of Anatomy and Neurobiology, Program in Neurosciences, University of Maryland School of Medicine, Baltimore, Maryland 21201

**Keywords:** circuit, dopamine, GABA, glomerular, inhibition, olfactory

## Abstract

The major inhibitory interneurons in olfactory bulb (OB) glomeruli are periglomerular cells (PGCs) and short axon cells (SACs). PGCs and SACs provide feedforward inhibition to all classes of projection neurons, but inhibition between PGCs and SACs is not well understood. We crossed Cre and GFP transgenic mice and used virally-delivered optogenetic constructs to selectively activate either SACs or GAD65cre-ChR2-positive PGCs while recording from identified GAD65cre-ChR2-positive PGCs or SACs, respectively, to investigate inhibitory interactions between these two interneuron types. We show that GAD65cre-ChR2-positive PGCs robustly inhibit SACs and SACs strongly inhibit PGCs. SACs form the interglomerular circuit, which inhibits PGCs in distant glomeruli. Activation of GAD65cre-ChR2-positive PGCs monosynaptically inhibit mitral cells (MCs), which complements recent findings that SACs directly inhibit MCs. Thus, both classes of glomerular inhibitory neurons inhibit each other, as well as OB output neurons. We further show that olfactory nerve input to one glomerulus engages the interglomerular circuit and inhibits PGCs in distant glomeruli. Sensory activation of the interglomerular circuit directly inhibits output neurons in other glomeruli and by inhibiting intraglomerular PGCs, may potentially disinhibit output neurons in other glomeruli. The nature and context of odorant stimuli may determine whether inhibition or excitation prevails so that odors are represented in part by patterns of active and inactive glomeruli.

## Significance Statement

In the olfactory bulb (OB) odors are initially processed by glomeruli. Glomeruli are surrounded by a complex network of excitatory and inhibitory neurons that transform sensory inputs into neural signals transmitted to subsequent olfactory networks. Glomerular excitatory and inhibitory neurons are richly interconnected and provide feedforward excitation and feedback inhibition to glomerular output neurons. Our findings add to the capacity and complexity of glomerular networks by showing that interglomerular and intraglomerular inhibitory circuits are reciprocally interconnected. These circuits could modulate signals sent to higher brain regions by directly inhibiting output neurons or by disinhibiting them when inhibitory circuits inhibit each other.

## Introduction

A fundamental principle in neuroscience is that the balance of excitation and inhibition determines neural circuit output signals. It is known that local circuit inhibitory neurons regulate excitatory output neurons but synapses between inhibitory neurons may disinhibit output neurons and play an important role in neural circuitry (Letzkus et al., 2015).

Odorants are transduced by olfactory sensory neurons, whose axons form the olfactory nerve that synapses onto excitatory output neurons—mitral cells (MCs), tufted cells (TCs), and external tufted cells (ETCs)—in olfactory bulb (OB) glomeruli. These excitatory pathways are strongly modulated by inhibitory interneurons. There are nearly as many inhibitory neurons in the glomerular layer (GL) as in the granule cell layer (Parrish-Aungst et al., 2007). GL inhibitory neurons act on the apical dendrites, whereas granule cells inhibit the lateral dendrites of output neurons. In addition, some deep short axon cells send inhibitory projections to glomeruli (Eyre et al., 2008; Burton et al., 2017).

The principal glomerular inhibitory neurons are GABAergic periglomerular cells (PGCs), and GABAergic-DAergic short axon cells (SACs; Kosaka and Kosaka, 2008; Kiyokage et al., 2010). PGCs are primarily “uniglomerular”, forming intraglomerular inhibitory circuits within a single glomerulus. In contrast, SACs are “multiglomerular”, forming interglomerular circuitry, which inhibit neurons, in other glomeruli, including MCs/TCs (Aungst et al., 2003; Shirley et al., 2010; Liu et al., 2013, 2016).

Together, intraglomerular and interglomerular inhibitory circuits regulate neuronal spike output to downstream networks (Shao et al., 2012). Inhibitory synapses between PGCs and SACs could, thus, significantly impact odor output signals, but little is known about potential interactions between these two inhibitory interneuron types. Electron microscope studies show symmetric synapses between glomerular cells (Price and Powell, 1970; Pinching and Powell, 1971; Toida et al., 1994) and inhibitory interactions among unidentified glomerular neurons have been reported (Murphy et al., 2005). This suggests there may be inhibitory interactions between interglomerular and intraglomerular circuits but the cell-type identity (PGCs or SACs), extent of potential interactions and their impact on glomerular output neurons are not well understood. To investigate these questions, we crossed Cre and GFP transgenic mouse lines and virally delivered optogenetic constructs to selectively activate either SACs or PGCs while recording from GFP-expressing PGCs or SACs, respectively.

## Materials and Methods

### Animals

Mice used in this study include a transgenic expressing green fluorescent protein (GFP) under the control of the glutamic acid decarboxylase-65 promoter (GAD65gfp; courtesy of Dr. Gabor Szabo, Hungary) or under the control of the tyrosine hydroxylase promoter (THgfp; courtesy of Dr. Kazuto Kobayashi, Japan), and transgenic mice expressing Cre recombinase under the glutamic acid decarboxylase-65 promoter (GAD65cre; Jax mice strain, B6.129.GAD65cre) or under the control of the tyrosine hydroxylase promoter (THcre; Jax mice strain, B6.Cg-Tg(Th-cre)1Tmd/J). Double-heterozygous mice were generated by crossing a heterozygote THgfp with a heterozygote GAD65cre, or a heterozygote GAD65gfp with a heterozygote THcre. Colonies of transgenic mice were maintained by breeding wild-type C57BL/6J female mice with a heterozygous male THgfp, THcre, or GAD65cre, or a wild-type B6CBAF1/J with a heterozygous male GAD65gfp. Approximately equal numbers of male and female mice were used in the experiments (total 25 animals). Analysis of the responses in each experiment did not show evidence of sex differences, thus results from male and female animals were pooled in the reported *n* for each experiment. All animal colonies and experimental procedures were performed in accordance with protocols approved by the Institutional Animal Care and Use Committee.

### Channelrhodopsin 2 expression

The optogenetic construct channelrhodopsin 2 (ChR2) was expressed in GAD65cre/THgfp or THcre/GAD65gfp using a Cre-inducible adeno-associated virus serotype2.9 (AAV2.9) carrying a fusion construct of ChR2 to the mCherry fluorescent protein (AAV-hSyn-hChR2(H134R)-mCherry; University of Pennsylvania Vector Core) injected into the GL of the medial side of each OB between postnatal weeks 3 and 4. Under deep anesthesia, the skull was exposed and a small hole (0.5 mm diameter) drilled over each OB at coordinates at 3.95 mm from bregma and 0.2 mm from midline. Injections were performed using a pulled glass micropipette (tip size 10–15 µm) and a pneumatic pressure injection apparatus (Picospritzer II, General Valve). AAV2.9 was injected into three points within the GL of the medial side of each bulb (depth: 2.0, 1.5, and 1.0 mm) at a rate of 0.1 μl/min for 5 min with a total volume of 0.5 μl per bulb. After 2–4 weeks for ChR2 mCherry fluorescent protein expression, acute horizontal OB slices were prepared for electrophysiology experiments.

### Slice preparation

Animals were anesthetized with saturated vapor isoflurane and the OBs surgically removed. The bulbs were immediately secured to a cutting platform and immersed in 4°C oxygenated sucrose-artificial CSF (sucrose ACSF) containing the following (in mm): 26 NaHCO_3_, 1 NaH_2_PO_4_, 3 KCl, 5 MgSO_4_, 0.5 CaCl_2_, 10 glucose, and 248 sucrose, equilibrated with 95% O_2_-5% CO_2_, pH 7.38. Horizontal slices (400 µm thick) were cut with a Leica VT1200s vibratome. Slices were incubated in oxygenated ACSF (in mm): 124 NaCl, 26 NaHCO_3_, 3 KCl, 1.25 NaH_2_PO_4_, 2 MgSO_4_, 2 CaCl_2_, and 10 glucose equilibrated with 95% O_2_-5% CO_2_, pH 7.38) at 30°C for 20–30 min and then at room temperature (22°C) in ACSF for at least 1 h before use. For recording, individual slices were transferred to a recording chamber and perfused with ACSF (as above) at a rate of 3 ml/min maintained at a temperature 30°C (Bipolar Temperature Controller). Target GFP-positive or MC cells were observed with a 40× water-immersion objective using an Olympus BX51W upright microscope equipped for near infrared differential interference contrast optics (Olympus Optical) and fluorescent excitation/barrier filters suitable for visualization of mCherry/GFP.

### Electrophysiology

Whole cell (current and voltage) patch-clamp recordings were performed using recording pipettes made from thick-wall borosilicate glass with filament (inner diameter: 0.75 mm; Sutter Instruments) pulled on a P-97 Flaming-Brown puller (Sutter). For current-clamp, the internal solution contained the following (in mm): 120 K-gluconate, 20 KCl, 10 HEPES, 2 MgCl_2_, 2 Mg2ATP, 0.2 Na3GTP, 0.1 BAPTA, and 0.02% Lucifer yellow, pH 7.3 adjusted with KOH and for voltage-clamp contained the following (in mm): 120 CsMeSO_4_, 10 QX-314, 10 HEPES, 1 MgCl_2_, 2.5 Mg_2_ATP, 0.2 Na_3_GTP, 0.1 BAPTA, 10 phosphocreatine, pH 7.3 adjusted with CsOH. Osmolarity for both solutions were in the range 287–295 mOsm. Recordings were discontinued if access resistance was >20 MΩ at the beginning of whole-cell recording with typical access resistances of 10-20 MΩ. Membrane capacitance (*C*_m_) for SACs was 6–10 pF and for PGCs were 5–8 pF. All data were acquired with pCLAMP 9 software using a MultiClamp 700A amplifier, digitized with a Digidata 1322A A/D board (Molecular Devices), low-pass filtered online at 2 kHz (voltage-clamp, sampling rate of 5 kHz) or 10 kHz (current-clamp, sampling rate of 40 kHz).

### Electrical and optical stimulation

Electrical stimulation of olfactory nerve axons was delivered by bipolar glass electrodes made from theta borosilicate tubes (Sutter Instruments). The electrodes were visually positioned 3–4 glomeruli rostral to the recording site. Isolated constant current pulses (100 µs, 20–100 µA) were triggered by a PG4000A Digital Stimulator (Cygnus Technologies). Optical stimuli (0.5–12 mW) were delivered from a 25 µm multimode optical fiber (0.1 numerical aperture, 7° beam spread; Thorlabs) coupled to a 150 mW, 473 nm, diode-pumped, solid-state laser (LWBL473083272) gated with a Uniblitz shutter (all light pulses were of 2 ms duration). Optical power delivered at the fiber tip was calibrated with a PM20A Power Meter (Thorlabs). The onset and duration of optical stimulation was measured during every experiment by splitting 1% of the laser beam out to a high speed (30 ns rise time) silicon photosensor (model 818-BB, Newport) and was recorded by the same MultiClamp 700A amplifier as the patch electrode.

### Data analysis

Data were analyzed with Clampfit 9.2 (Molecular Devices). Statistical analysis and graphical presentation was performed with Origin (OriginLab). Statistical significance of population responses was calculated by using Student’s *t* test (comparing two groups) or ANOVA with Bonferroni *post hoc* (comparing >2 groups). Graphs and plotting were created with Origin 8.5 and CorelDraw 17.

### Drugs and chemicals

APV (50 m), NBQX (disodium salt, 10 µm), gabazine (GBZ; SR95531, 10 µm), 8-bromo-2,3,4,5-tetrahydro-3-methyl-5- phenyl-1H-3-benzazepin-7-ol (SKF83566) hydrobromide (10 µm), (2S)-3-[[(1S)-1-(3,4-Dichlorophenyl) ethyl]amino-2ydroxypropyl] (phenylmethyl) phosphinic acid hydrochloride (CGP55845, 10 µm), were purchased from Tocris Cookson. All other chemicals were purchased from Sigma-Aldrich. All drugs were bath applied by diluting in ACSF at the above-indicated doses unless otherwise stated. Drugs enter the recording chamber 45 s after switching fluid lines. To ensure adequate drug access to the slices, all recordings occurred no sooner than 5 min after fluid switch.

## Results

The two major classes of glomerular inhibitory neurons are PGCs and SACs, which form intraglomerular and interglomerular circuits, respectively. The existence of spontaneous and evoked IPSCs in both cell types (Murphy et al., 2005; Liu et al., 2015; Brill et al., 2016) and electron microscopy evidence for synapses between glomerular inhibitory neurons (Price and Powell, 1970; Pinching and Powell, 1971; Toida et al., 1994) suggests that PGCs and SACs are synaptically interconnected, but it is not known whether both are involved. To explore this, and to see if intraglomerular and interglomerular circuits reciprocally inhibit each other, we used a genetic-ChR2 strategy to assess the actions of PGCs and SACs on one another.

### SACs directly inhibit PGCs

**Figure 1.**
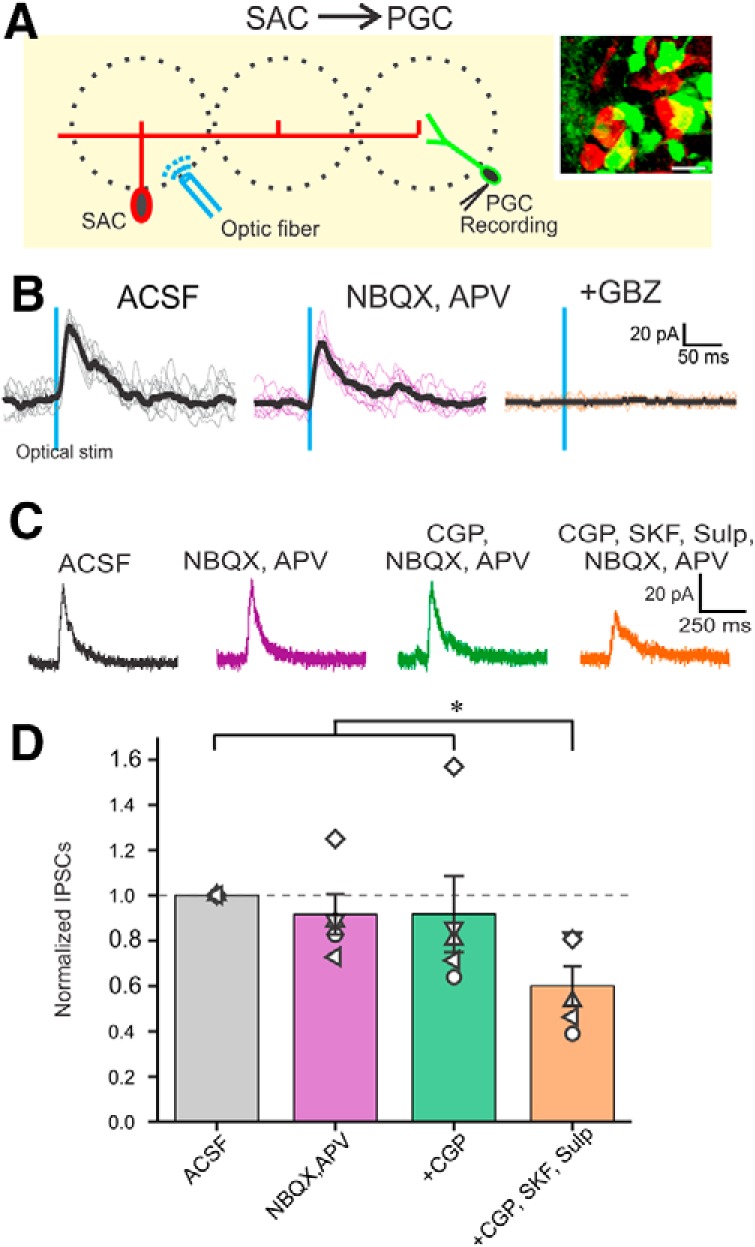
Activation of ChR2-SACs evoked inhibitory response in GAD65gfp PGCs. ***A***, Schematic diagram showing the experimental design of optical stimulation of ChR2-SACs and recording from PGCs. Inset, GAD65gfp (green)- and ChR2-mCherry-labeled THcre neurons in the GL. Scale bar, 10 µm. ***B***, Voltage-clamp recording of a PGC held at 0 mV, in ACSF, 10 µm NBQX, and 50 µm APV, and in further addition of 10 µm GBZ. ***C***, Optically evoked IPSCs from a PGC in ACSF (left), NBQX/APV (middle left), further addition of CGP55845 (CGP; middle right), and further addition of D1/D2 blockers SKF83566 and sulpiride (SKF and Sulp, respectively; right). ***D***, IPSC amplitude from eight PGCs normalized to ACSF in the presence of NBQX/APV, further addition of CGP55845, and further addition of D1/D2 blockers SKF83566 and sulpiride. NBQX/APV/CGP/SKF/Sulp was significantly different (*p < 0.05) from ACSF, NBQX/APV, and NBQX/APV/CGP. There were no significant differences between ACSF, NBQX/APV, and NBQX/APV/CGP.

Activation of the SAC interglomerular circuit inhibits mitral and tufted cell excitatory neurons in distant glomeruli (Aungst et al., 2003; Shirley et al., 2010; Liu et al., 2013, 2016; Whitesell et al., 2013; Banerjee et al., 2015). Here, we asked do SACs inhibit PGCs to impact intraglomerular circuitry? For this, we crossed mice expressing GFP under the control of the GAD65gfp mice (a PGC marker; Shao et al., 2009) with mice expressing Cre-recombinase under the control of the TH promoter (THcre mice); TH is expressed by all SACs (Kosaka and Kosaka, 2008; Kiyokage et al., 2010). The OBs of the resulting offspring were injected with ChR2-mCherry AAV constructs resulting in GFP-positive PGCs and ChR2-expressing SACs. As a result, optical activation of TH-ChR2 cells selectively excited SACs. PGCs were clamped at 0 mV to optimize detection of IPSCs (Fig. 1*A*) while activating SACs located at least three glomeruli distant (>200 μm). PGCs (and SACs) can receive direct olfactory nerve input or indirect input via ETCs (Shao et al., 2009) assessed by the presence of spontaneous bursts of EPSCs. We did not observe any difference between cells of the direct/indirect olfactory nerve input pathway and data below are pooled.

**Figure 2.**
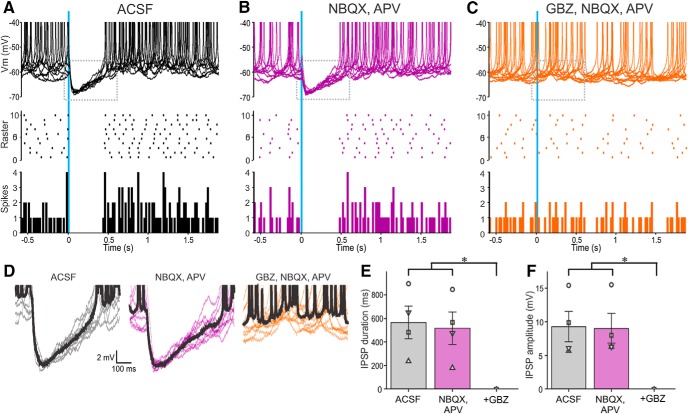
Activation of ChR2-SACs inhibits PGC output. ***A*–*C***, Ten superimposed current-clamp traces showing PGC responses to brief optical stimulation (vertical blue bar) of ChR2-SACs in (***A***) ACSF, (***B***) 10 µm NBQX, and 50 µm APV and (***C***) with further addition of 10 µm GBZ. The raster plot (***A***–***C***) in the middle is from the cell in the top . The bottom (***A***–***C***) shows a population PSTH plot representing averaged spike data in four PGCs. ***D***, Traces showing IPSPs evoked by optical stimulation of ChR2-SACs from the dotted rectangles in the corresponding top panels in ***A***–***C***. The thick black trace represents the average of the 10 traces. ***E***, Population data from four PGCs showing the effects of stimulation in ACSF, NBQX/APV, or NBQX/APV plus GBZ on IPSP duration. NBQX/APV/GBZ was significantly different (*p < 0.01) from the groups ACSF and NBQX/APV. There were no significant differences between the groups ACSF and NBQX/APV. ***F***, Population data from four PGCs showing the effects of stimulation in ACSF, NBQX/APV, or NBQX/APV plus GBZ on IPSP amplitude. NBQX/APV/GBZ was significantly different (*p < 0.01) from the groups ACSF and NBQX/APV. There were no significant differences between the groups ACSF and NBQX/APV.

Selective activation of SACs evoked robust IPSCs in all PGCs recorded. IPSCs were short latency 1.89 ± 0.04 ms (range 1.76–1.98 ms; *n* = 5) and low jitter 119 ± 15 µs (range 89–169 µs; *n* = 5) consistent with monosynaptic inhibition. The peak amplitude of the IPSC was 10–20 pA following a brief, single optical activation of SACs (473 nm, 2 ms pulse). IPSCs were abolished by gabazine but impervious to glutamate blockers, which obviates excitatory circuit actions (35.74 ± 8.3 pA in ACSF and 33.94 ± 8.78 pA in NBQX/APV; no significant difference; *n* = 5; Fig. 1*B*). GAD65gfp neurons had a resting membrane potential of −55.25 ± 2.05 mV (*n* = 5) consistent with previous reports (Shao et al., 2009), with no significant difference in spontaneous spiking activity between ACSF and NBQX/APV (2.5 ± 0.7 spikes/s in ACSF; 2.2 ± 0.7 spike/s in NBQX/APV; no significant difference; *n* = 5). Because GAD65gfp neurons have a high input resistance of 673 MΩ (Shao et al., 2009), an optically evoked IPSC should generate sufficient hyperpolarization to inhibit spiking. Indeed, optical activation of SACs also completely eliminated spontaneous PGCs spiking (Fig. 2*A*,*D*). Conceivably, this could be because of indirect effects as SACs inhibit ETCs, which might reduce excitation of PGCs. Arguing against this, however, addition of NBQX/APV, which blocks ETC→PGC excitation did not alter SAC→PGC IPSPs or spontaneous PGC spiking (*n* = 4; Fig. 2*B*,*D*–*F*). DA increases *I*_h_ in ETCs causing rebound excitation, increasing ETC excitatory drive to PGCs (Liu et al., 2013). However, addition of DA D1 and D2 receptor blockers reduced SAC-evoked IPSCs in PGCs (as described in the section “GABAB and DA in glomerular inhibitory circuits”). Together these results indicate that SACs inhibit PGCs in distant glomeruli by direct activation of GABA_A_ receptors.

### On activity engages interglomerular inhibition of intraglomerular circuits

**Figure 3.**
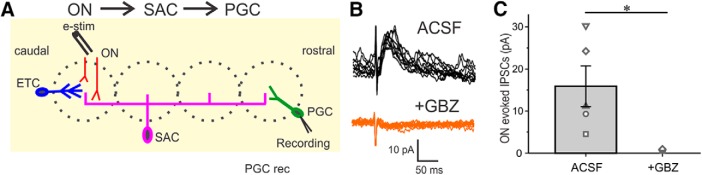
E-stimulation of the olfactory nerve evokes PGC inhibitory responses in distant glomeruli. ***A***, Schematic diagram showing the experimental design of electrical stimulation to the olfactory nerve layer (ONL) and PGC recording in a distant glomerulus. ***B***, Nerve stimulation evokes IPSCs in a PGC held at 0 mV four glomeruli away from the stimulation site. Responses are abolished by the addition of 10 µm GBZ. ***C***, Population data showing olfactory nerve stimulation evoked IPSC amplitude from five PGCs at least four glomeruli from the stimulation site in ACSF and in 10 µm GBZ. Statistical significance *p < 0.01.

Can interglomerular inhibition of intraglomerular circuits be elicited by ON input? To explore this, we recorded from GAD65gfp+ cells while electrically stimulating the olfactory nerve 3–4 glomeruli caudal to the recorded cell. As ON axons do not “loop-back” rostrally over this distance, this experimental configuration activates the interglomerular circuitry and obviates ON synapses on the recorded PGC. Stimulating ON in this configuration activated interglomerular projections and evoked robust IPSCs in the recorded cell but did not evoke short latency EPSCs, confirming the absence of direct ON synapses. Interglomerular-evoked IPSCs had a latency of 5.45 ± 0.21 ms (range from 4.83 to 5.93 ms) with jitter of 825 ± 190 µs (range from 314 to 1256 µs) consistent with a polysynaptic ON→SAC→PGC interglomerular circuit. IPSCs were completely blocked by GBZ (15.89 ± 4.85 pA in ACSF vs 0 ± 0.32 in GBZ; *p* < 0.01; *n* = 5; Fig. 3). These findings show that sensory signals can activate the interglomerular circuit to suppress intraglomerular PGCs in distant glomeruli.

### PGCs inhibit MCs and SACs

MCs receive monosynaptic ON input, which is augmented by di-synaptic excitatory inputs from ETCs. These excitatory currents generate long-lasting depolarizing (LLD) excitation in MCs (Carlson et al., 2000; De Saint Jan et al., 2009; Najac et al., 2011; Gire et al., 2012; Shao et al., 2012). The onset of this compound EPSC is followed by an IPSC with latency 6.6 ms and jitter 432 µs (Shao et al., 2012). This inhibitory current shortens the duration of the LLD and is blocked by intraglomerular application of gabazine (Shao et al., 2012), indicating that it is because of glomerular GABAergic interneurons. Although the inhibition is generally attributed to PGCs (Gire and Schoppa, 2009; Shao et al., 2012, 2013; Najac et al., 2015), SACs also generate potent monosynaptic inhibition of MCs (Aungst et al., 2003; Shirley et al., 2010; Liu et al., 2013, 2016; Whitesell et al., 2013; Banerjee et al., 2015). Indeed, the only evidence for monosynaptic PGC→MC inhibition is a report based on a small sample of PGC→MC paired recordings (Najac et al., 2015).

**Figure 4.**
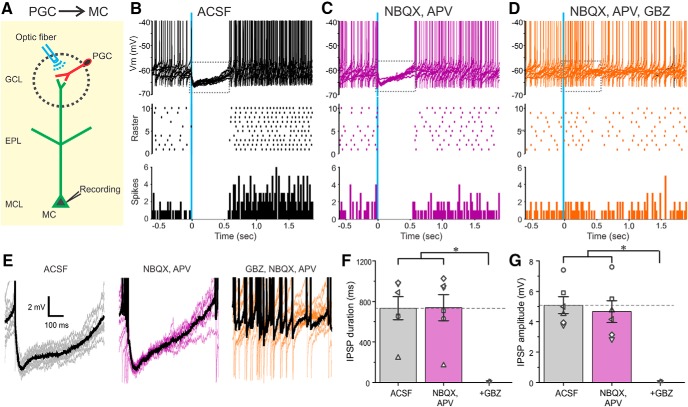
PGCs inhibit MC output. ***A***, Schematic diagram showing the experimental design of optical stimulation of ChR2-PGCs and MC recording. ***B***–***D***, Ten superimposed current-clamp sweeps showing long-lasting MC inhibition to brief optical stimulation (vertical blue bar) of CHR2-PGCs in ACSF (***B***), in the presence of 10 µm NBQX and 50 µm APV (***C***). Addition of 10 µm GBZ blocks the inhibition of MCs (***D***). The raster plot (***B***–***D***) in the middle is from the cell in the top . The bottom (***B***–***D***) shows a population PSTH of averaged spike data from six MCs. ***E***, Traces showing IPSPs evoked by optical stimulation of ChR2-PGCs expanded from the dotted rectangles in the corresponding top in ***B***–***D***. The thick black trace represents the average IPSP of the 10 sweeps. ***F***, Population data from six MCs shows the effect of PGC stimulation on MCs in ACSF, NBQX, and APV, or NBQX/APV plus GBZ on IPSP duration. NBQX/APV/GBZ was significantly different (*p < 0.0001) from the groups ACSF and NBQX/APV. There were no significant differences between the groups ACSF and NBQX/APV. ***G***, Population data from six MCs shows the effect of PGC stimulation on MCs in ACSF, NBQX, and APV, or NBQX-APV plus GBZ on IPSP amplitude. NBQX/APV/GBZ was significantly different (*p < 0.0001) from the groups ACSF and NBQX/APV. There were no significant differences between the groups ACSF and NBQX/APV.

To seek additional evidence, ChR2 was expressed in GAD65cre neurons by AAV injection into the GL. ChR2 was activated by laser light (473 nm, 2 ms pulses) delivered via 25 µm fiber optic placed over a glomerulus containing the dendrite of a patched, Lucifer yellow filled, MC (Fig. 4*A*). PGCs preferentially express GAD65 (Parrish-Aungst et al., 2011), but this isoenzyme is also expressed by some SACs (Parrish-Aungst et al., 2007), which may also have been activated. Arguing against this, however, is the fact that in all GAD65-ChR2 cell→MC experiments inhibitory currents were evoked only when the optical fiber targeted the glomerulus containing the recorded MC’s apical dendrite. When the fiber was moved to nearby glomeruli responses were not evoked. This indicates that contamination by GAD65-expressing SACs was negligible (see Discussion).

Activation of PGCs generated hyperpolarization in SACs that was blocked by gabazine, but not glutamate receptor blockers (5.08 ± 0.56 mV in ACSF and 4.66 ± 0.71 mV in NBQX/APV; no significant difference; *n* = 6; Fig. 4*E*,*F*; resting membrane potential −57.2 ± 2.33 mV). IPSP latencies were 2.23 ± 0.06 ms (range 1.96–2.38 ms; *n* = 6) with jitter of 191.36 ± 15.55 µs (range 139.39-231.13 µs; *n* = 6) consistent with PGC→MC monosynaptic inhibition. Activation of PGCs also caused immediate, sustained inhibition of spontaneous MCs spiking (*n* = 6; Fig. 4*B*). Inhibition was long lasting (733 ± 115 ms in ACSF and 738 ± 130 ms in NBQX/APV; no significant difference; *n* = 6; Fig. 4*E*,*F*), exceeding the brief 2 ms optical activation of PGCs. This is consistent with previous reports that MC intrinsic membrane properties prolong the duration of synaptic inhibition (Liu et al., 2016). Together, the preceding results demonstrate that both PGCs and SACs directly inhibit MCs and that MC intrinsic properties prolong these inhibitory actions (Liu et al., 2016). Thus, both intraglomerular and interglomerular inhibitory circuits potently regulate MCs.

**Figure 5.**
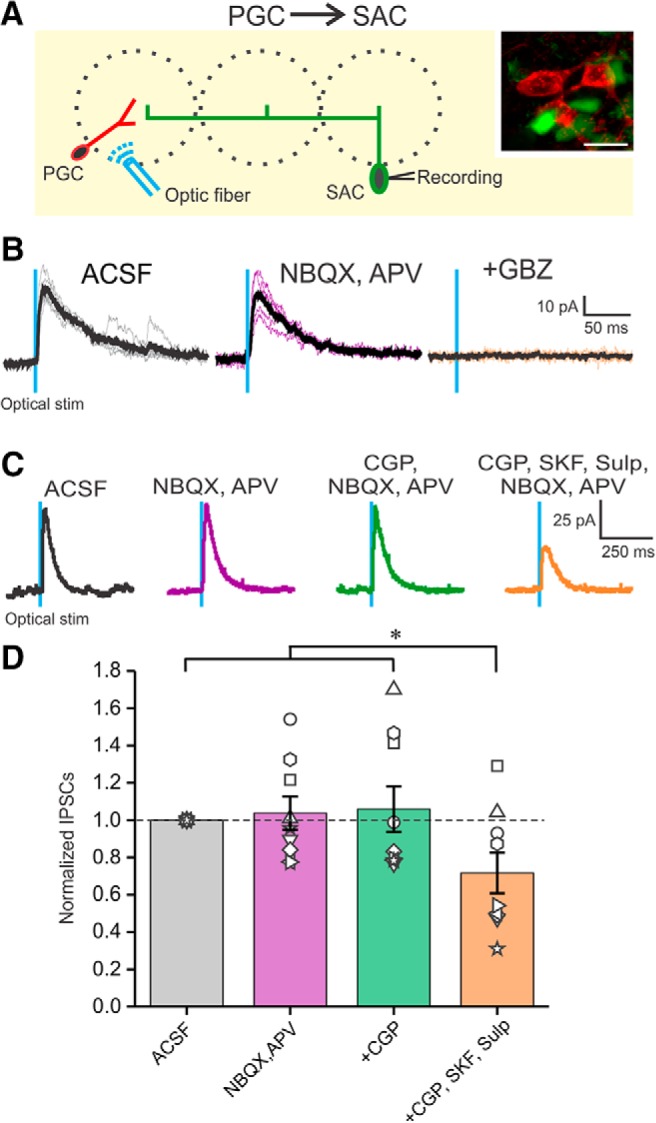
Activation of ChR2-PGCs evokes inhibitory responses in THgfp-positive SACs. ***A***, Schematic diagram showing the experimental design of optical stimulation of ChR2-PGCs and recording from SACs. Inset, THgfp- (green) and ChR2-mCherry-labeled GAD65cre neurons in the GL. Scale bar, 10 µm. ***B***, Voltage-clamp recording from an SAC held at 0 mV exhibiting optical evoked IPSC in ACSF (left), in NBQX/APV (middle), and in further addition of GBZ (right). The thick black trace represents the average IPSC of 10 sweeps. ***C***, Optically evoked IPSCs from SAC in ACSF (left), NBQX/APV (middle left), further addition of CGP55845 (CGP; middle right), and further addition of D1/D2 blockers SKF83566 and sulpiride (SKF and Sulp, respectively; right). ***D***, IPSC amplitude from nine SACs normalized to ACSF in the presence of NBQX/APV, further addition of CGP55845, and further addition of D1/D2 blockers SKF83566 and sulpiride. NBQX/APV/CGP/SKF/Sulp was significantly different (*p < 0.05) from ACSF, NBQX/APV, and NBQX/APV/CGP. There were no significant differences between ACSF, NBQX/APV, and NBQX/APV/CGP.

To determine whether PGCs also inhibit SACs, GAD65cre mice were crossed with mice expressing GFP under control of the TH promoter. ChR2 was introduced into GAD65 neurons by AAV injection. As noted, some (∼20%) SACs express GAD65 and thus could provide SAC→SAC inhibition. However, as there were no responses to interglomerular activation in the PGC→MC experiments (above), SAC contamination appears functionally negligible, thus ChR2 responsive GAD65-expressing cells are assumed to be mainly PGCs. Optical activation of PGCs evoked robust IPSCs in all recorded THgfp^+^ SACs (Fig. 5*A*; *n* = 5). IPSCs were short latency 1.85 ± 0.05 ms (range 1.77–2.01 ms; *n* = 5) with low jitter 142 ± 38 µs (range 43–274 µs; *n* = 5) consistent with monosynaptic PGC→SAC inhibition. IPSCs were abolished by gabazine (10 µm GBZ), but unaltered by ionotropic glutamate receptor block (29.8 ± 14.66 pA in ACSF vs 27.8 ± 14.78 pA in NBQX/APV, no significant difference; *n* = 5; Fig. 5*B*).

**Figure 6.**
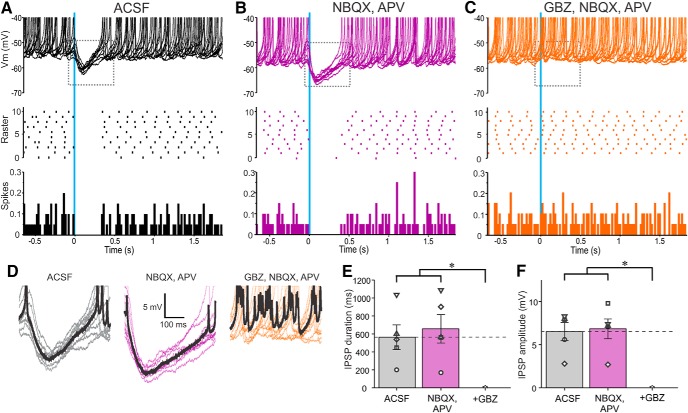
Activation of ChR2-PGCs inhibits SAC output. ***A***–***C***, Ten superimposed current-clamp traces showing SAC responses to brief optical stimulation (vertical blue bar) of ChR2-PGCs in (***A***) ACSF, (***B***) 10 µm NBQX, and 50 µm APV and (***C***) with further addition of 10 µm GBZ. The raster plot (***A***–***C***) in the middle is from the cell in the top . The bottom (***A***–***C***) shows a population PSTH plot representing averaged spike data from five SACs. ***D***, Traces showing IPSPs evoked by optical stimulation of ChR2-PGCs expanded from the dotted rectangles in the corresponding top panels in ***A***–***C***. The thick black trace represents the average IPSP of the 10 traces. ***E***, Population data from five SACs showing the effects of PGC stimulation on SACs in ACSF, NBQX/APV, or NBQX/APV plus GBZ on IPSP duration. NBQX/APV/GBZ was significantly different (*p < 0.01) from the groups ACSF and NBQX/APV. There were no significant differences between the groups ACSF and NBQX/APV. ***F***, Population data from five SACs showing the effects of PGC stimulation of SACs in ACSF, NBQX/APV, or NBQX/APV plus GBZ on IPSP amplitude. NBQX/APV/GBZ was significantly different (*p < 0.001) from the groups ACSF and NBQX/APV. There were no significant differences between the groups ACSF and NBQX/APV.

SACs have spontaneous, as well as ON- and ETC-evoked action potentials. Brief (2 ms) optical activation of PGCs evoked a 6.52 ± 1.05 mV IPSP lasting for 564 ± 136 ms (*n* = 5; Fig. 6*A*,*D*–*F*) and completely eliminated spontaneous SAC spiking. To isolate SACs from ETC glutamatergic drive NBQX and APV were added to the bath. Consistent with ETC excitation of SACs (Hayar et al., 2004), this reduced SAC spontaneous spiking by ∼7.2% (4.8 ± 1.1 spike/s in ACSF, 4.5 ± 1.5 spikes/s in NBQX/APV; no significant difference; *n* = 5; Fig. 6*A*,*B*). However, PGC activation still evoked a robust IPSP and eliminated residual spontaneous SAC spiking (*n* = 5; Fig. 6*B*–*F*). This shows that PGCs activate postsynaptic GABA_A_ receptors to directly inhibit SACs.

### GABA_B_ and DA in glomerular inhibitory circuits

Glomerular circuitry also contains GABA_B_ receptors that can act at presynaptic and/or postsynaptic GABA_B_ receptors to modulate glomerular circuits (Wachowiak and Cohen, 1999; Aroniadou-Anderjaska et al., 2000; Ennis et al., 2001; McGann et al., 2005; Vucini et al., 2006; Karpuk and Hayar, 2008; Vaaga et al., 2017). Since both PGCs and SACs release GABA, optically evoked responses in the corresponding neuron could be influenced by GABA_B_ receptors. However, addition of a GABA_B_ blocker CGP55845 (10 µm) had no effect on the duration, amplitude, or latency/jitter of the SAC→PGC evoked IPSCs (*n* = 5; Fig. 1*C*,*D*) or the PGC→SAC evoked IPSCs (*n* = 5; Fig. 5*C*,*D*). SACs release DA as well as GABA (Liu et al., 2013). DA tonically inhibits presynaptic terminals of the olfactory nerve (Ennis et al., 2001) and modulates ETC membrane properties (Liu et al., 2013). Thus, tonic DA release may modulate SAC-evoked PGC responses and/or PGC evoked SAC responses. To test this D1/D2 blockers (10 µm SKF83566, 100 µm sulpiride) were added along with glutamate receptor blockade. D1 and D2 blockers decreased SAC→PGC IPSC amplitude by ∼40% (22.27 ± 2.16 pA in ACSF and 12.73 ± 1.14 pA in D1/D2 blockers; *n* = 5, *p* < 0.05; Fig. 1*C*,*D*) and PGC→SAC IPSC amplitude by 23% (48.6 ± 11.7 pA in ACSF and 37.4 ± 11.7 pA in D1/D2 blockers; *p* < 0.05; *n* = 9; Fig. 6*C*,*D*). This tonic action of DA, which enhances inhibition at the SAC→PGC and the PGC→SAC synapses, may be presynaptic or postsynaptic or both.

## Discussion

Glomeruli regulate odor signals at the initial stage of synaptic integration in the olfactory system (for review, see Ennis et al., 2014). Throughput is initiated by olfactory nerve excitation of output neurons, MCs/TCs, and is strongly modulated by glomerular inhibitory circuits. The major glomerular inhibitory neurons are GABAergic PGCs and GABAergic/DAergic SACs. They provide presynaptic inhibition of ON synapses and postsynaptic inhibition of MCs/TCs. PGCs provide mainly intraglomerular inhibition while SACs mediate both intraglomerular and interglomerular inhibition of neurons in other glomeruli.

PGCs and SACs receive mono- (type 1) or polysynaptic (type 2) (Toida et al., 1994, 1998, 2000; Kosaka et al., 1995, 1997; Shao et al., 2009; Kiyokage et al., 2010) excitatory inputs from olfactory nerve terminals (Shao et al., 2009; Liu et al., 2013) and monosynaptic excitation from OB projection neurons (Hayar et al., 2004). Together, glomerular inhibitory and excitatory neurons form complex, heterogeneous circuits. Here we show that PGCs and SACs inhibit each other, as well as glomerular output neurons. This adds to the processing richness of glomerular networks and demonstrates that glomerular inhibitory circuits potently modulate neural processing of odorant stimuli.

### PGCs inhibit MCs

MCs receive IPSCs from neurons in glomerular circuits (Shao et al., 2012). Although this is presumed to come from PGCs, there is surprisingly little, direct evidence for PGC→MC monosynaptic inhibition, primarily a small sample of paired PGC→MC recordings (Najac et al., 2015). To seek additional evidence, we expressed ChR2 in GAD65cre cells (Parrish-Aungst et al., 2007). This evoked robust IPSCs in MCs that were completely blocked by a GABA_A_ receptor antagonist. IPSCs had latencies (<2 ms) and jitter (<200 µs) consistent with a monosynaptic connection. Moreover, they were impervious to glutamate receptor blockers, thus obviating excitatory circuit effects. GAD65 is expressed by all PGCs and some SACs (∼20%; Parrish-Aungst et al., 2007). Thus some of the inhibitory currents might be attributable to SACs. However, inhibition was evoked only when the 25 µm fiber targeted the glomerulus containing the dendritic tuft of the recorded MC. Activation of GAD65-ChR2 cells 2–4 glomeruli distant did not evoke any responses in MCs.

There are several possible reasons why GAD65-expressing SACs may not contribute to MC inhibition. First, the subset of GAD65-expressing SACs (Parrish-Aungst et al., 2007) may be too few in number to generate detectable IPSCs in MCs. Second, some SACs innervate only 4–7 nearby glomeruli. These “oligoglomerular” SACs (Kiyokage et al., 2010) may correspond to the recently reported short-range projecting DA “axon-initial-segment negative” SACs (Galliano et al., 2018). We stimulated at least three glomeruli distant, which may have been beyond the processes of these SACs even if they did express GAD65-ChR2. Finally, DA neurons are continuously generated and migrate into glomeruli throughout adult life (Luskin, 1993; Lois and Alvarez-Buylla, 1994; Kohwi et al., 2005; De Marchis et al., 2007; Galliano et al., 2018). Immature DA cells express TH promoter activity before expression of TH protein or GAD enzymes (Baker et al., 2001; De Marchis et al., 2004; Plachez and Puche, 2012). It is conceivable, therefore, that these immature cells transiently express the GAD65 transgene, but do not yet contribute significantly to interglomerular inhibition. Thus, while we cannot exclude a small contribution of GAD65^+^ SACs to MC inhibition, the parsimonious conclusion is that it is mainly, if not exclusively, because of PGCs.

### Mutual PGC-SAC inhibition

PGCs and SACs are involved in diverse glomerular circuit functions. For example, GABA_B_ receptors are expressed on olfactory nerve terminals (Bonino et al., 1999; Panzanelli et al., 2004) and ON excitation of PGCs and SACs may evoke GABA release to cause presynaptic inhibition of sensory input (Wachowiak and Cohen, 1999; Aroniadou-Anderjaska et al., 2000; Murphy et al., 2005; Wachowiak et al., 2005; Pirez and Wachowiak, 2008; Shao et al., 2009). Activation of PGCs and SACs evoke IPSC/Ps in ETCs, as well as MCs/TCs (present study (Aungst et al., 2003; Hayar et al., 2005; Murphy et al., 2005; Whitesell et al., 2013; Banerjee et al., 2015; Najac et al., 2015; Liu et al., 2016). ON terminals have DA D2 presynaptic receptors and tonic and evoked DA release by SACs inhibits sensory inputs (Koster et al., 1999; Wachowiak and Cohen, 1999; Ennis et al., 2001). SACs form the interglomerular circuit, which synapse onto neurons in neighboring and distant glomeruli (Aungst et al., 2003; Kosaka and Kosaka, 2008; Kiyokage et al., 2010; Shirley et al., 2010; Whitesell et al., 2013; Banerjee et al., 2015; Galliano et al., 2018). SACs co-release GABA and DA, which acts postsynaptically on M/TCs and ETCs (Borisovska et al., 2013; Liu et al., 2013, 2016). PGCs form mainly intraglomerular circuits that act in a single glomerulus. Thus, PGCs and SACs have numerous presynaptic and postsynaptic inhibitory targets and play multiple roles shaping glomerular input-output signal processing.

Here, we asked whether there are synaptic interactions between PGCs and SACs. Our findings show that GABA_A_ receptor-dependent IPSC/Ps were elicited at both SAC→PGC and PGC→SAC synapses. IPSCs in both circuits had short latency and low jitter (latency <2 ms, jitter <200 µs) indicative of monosynaptic connections. SAC→PGC inhibition was evoked by activation of distant glomeruli, showing that interglomerular SAC projections target PGCs, as well as ETCs and MCs (Aungst et al., 2003; Shirley et al., 2010; Borisovska et al., 2013; Liu et al., 2013, 2016; Whitesell et al., 2013; Banerjee et al., 2015). Our findings further show that interglomerular connectivity is functionally relevant, as activation of ON inputs to one glomerulus evoke IPSC/Ps in PGCs of distant glomeruli.

Olfactory sensory input is dynamic, regulated by the respiratory and sniffing behavior of the animal, generating episodic excitatory input to the glomerular circuitry. In this study we show that olfactory nerve input, in addition to excitatory-inhibitory neuron synapses and the reciprocal excitatory-excitatory connections between ETCs/MCs (Hayar et al., 2004; De Saint Jan et al., 2009; Najac et al., 2011), can also elicit activity within the robust inhibitory to inhibitory circuits during olfactory sensory processing. Inhibitory connections between PGCs and SACs may regulate glomerular circuit dynamics in odor processing. The interglomerular circuit could generate opposing actions on MCs in different, distant glomeruli: SACs directly inhibit MCs and ETCs reducing their output (Liu et al., 2016) but they also inhibit PGCs, which could disinhibit ETCs and MCs, thus increasing their excitability. Synchronous interglomerular network activation (Aungst et al., 2003; Shirley et al., 2010; Liu et al., 2016) generates net inhibition of MCs. *In vivo*, however, odorant stimuli elicit unique patterns of suppression and excitation in subsets of spatially intermingled glomeruli (Economo et al., 2016). Odorant activation of the interglomerular circuit may generate net suppression of some glomerular subsets and net activation of others depending on the nature and context of the odorant stimulus. Reciprocal PGC-SAC inhibition may contribute to a network dynamic that determines the balance of glomerular excitation and inhibition in response to different odorants, including potentially modulating temporal dynamics in the circuit. How these networks are modulated by input frequency, as in sniffing, remains to be explored.

## Synthesis

Reviewing Editor: Zoltan Nusser, Institute of Experimental Medicine Decisions are customarily a result of the Reviewing Editor and the peer reviewers coming together and discussing their recommendations until a consensus is reached. When revisions are invited, a fact-based synthesis statement explaining their decision and outlining what is needed to prepare a revision will be listed below. The following reviewer(s) agreed to reveal their identity: David Gire.

This is well-organized MS based on experiments that appear to be well executed. The authors combined reporters and Chr2-positive cell types that enabled them to perform specific recording and activation of PGC and SAC cell types. Both reviewers and I found that there are issues regarding the statistics in the MS. For example, there is no indication of what test was used or the statistical significance for the comparisons indicated by asterisks in figures 1 and 5. Tests should also be conducted and reported for data in figures 2, 3, 4 and 6. Furthermore, the paragraph at bottom of page 7 to the top of page 8 needs more statistics included. While some points contain n-values and statistical analyses others are just stated without any statistical support. There is also a strange comment that reads as if a note to one of the authors was accidentally left in the manuscript. There are a number of additional issues raised by the reviewers that needs to be addressed prior to publication.

The manuscript would benefit from the inclusion of figure panels showing the expression of the injected virus in each mouse line. A panel showing this should be added to figure 1 for the injections in TH-cre mice, and to figure 5 showing the injections in the GAD65-cre mice.

Were any of the recorded cells recovered to show their morphology? An example of each would be informative.

What were the access resistances and whole cell capacitances of the cells? Please include this data in the manuscript.

How long were the drugs washed into the bath before the traces shown in the figures were recorded? Please include this data in the manuscript.

In figure 1, panel D, the legend states n = 8, but I can only see 5 different datapoints - please clarify and correct this.

What is the spontaneous activity in the PGCs caused by? Is this due to any current applied to the cell in current clamp mode? What is the PGC resting potential? Please include this data in the manuscript.

What is responsible for the long (600-800 ms) pauses in firing upon brief (2 ms) optical illumination? Is this due to membrane dynamics, similar to MCs (Liu et al 2016)? Is this the mechanism of the effect observed in figure 4? Can the authors provide any data for this? For example, does a brief depolarization alone cause a cessation of firing for 600-800 ms in PGCs? Please include this data in the manuscript.

Similarly, is this also the case for SACs? What are their spontaneous firing rates? How much current is injected? What is the resting membrane potential? Please include this data in the manuscript.

Please comment on the reason for the application of both D1 and D2 receptor blockers at the same time. These receptors have counteracting effects on intracellular signalling cascades, and their combined inhibition may be hard to interpret. What data is there regarding whether SACs and/or PGCs express dopamine receptors? How could their expression affect the simultaneous application of both blockers?

Did the authors observe any differences in the responses of PGCs and/or SACs, considering the diversity of these cells e.g. in terms of their direct or indirect OSN inputs? Please include this data in the manuscript.

What are the connectivity (convergence/divergence) ratios and connection/synapse strengths between the two cell types? These data would be relevant and add a quantitative dimension to the interconnectivity data of the two cells types. It would also be useful for computational modelling of the complex interactions in the olfactory glomerulus.

The title is not completely appropriate and should be revised: “Excitation-Inhibition Balance” is only indirectly related to the evoked currents or pauses in action potential firing reported in the manuscript. To be more informative, the title should include the words “mouse olfactory bulb”.

The last paragraph of the discussion is speculative and not very useful - it should be changed to focus on the in vivo functional implications of the data in the manuscript. Odor processing in vivo is a dynamic process, involving timing and oscillatory rhythms, and the data regarding excitatory and inhibitory signalling should be discussed in this regard.

There are a number of errors in the manuscript that should be corrected:

In the Abstract: In the first line, the abbreviation for periglomerular cells is an error (PSCs - should be PGCs).

It would help the clarity of the manuscript if hyphens could be used after the abbreviations (SACs, PGCs, GAD65cre) in the third and sixth sentences.

In the significance statement, the fourth sentence does not need the word ‘we’.

In the Introduction, at the end of the second paragraph, the citation of (Presser & Strowbridge, 2006) is not appropriate - this paper presents data on Blanes cells that innervate the GCL Granule cells, and does not show data on GLdSACs or connections with glomeruli. A more appropriate citation would be Eyre MD, Antal M, Nusser Z (2008) Distinct deep short-axon cell subtypes of the main olfactory bulb provide novel intrabulbar and extrabulbar GABAergic connections. J Neurosci 28:8217-8229.

Methods: Slice preparation: Was the objective a 40x water-immersion objective?

Please can the authors add details of the equipment used for the stereotaxic injection - was a Hamilton syringe used?

Please state the duration of the light pulses in the methods - it is mentioned in the Results section with respect to figures 4 and 6, but no data is given regarding figure 2.

Results: SACs directly inhibit PGCs:

First paragraph: The third sentence should be revised - what is meant by “Offspring olfactory bulbs..” ?

Second paragraph: The third sentence states that “The peak amplitude of the IPSC was 10-20 ms...” Should this be 10-20 pA? Please also state the duration of the optical stimulus here.

Second paragraph: Penultimate sentence - a reference to a figure seems to be missing, or preliminary - “(below???)”

Results: PGCs inhibit MCs and SACs: In the second sentence, the reference “ De Saint et al., 2009 ” is incorrect, and should be “De Saint Jan et al., 2009”. Please correct the entry in the Reference list as well.

Results: GABAB and DA in glomerular inhibitory circuits; 7th sentence - please use the word “blockade” instead of “blocks”.

Discussion: PGCs inhibit MCs:

1st paragraph, 3rd sentence - please change to “... we expressed ChR2 in GAD65cre cells...”

penultimate sentence - please change “with” to “containing”.

2nd paragraph, second sentence, please add the word “be” - “...may be too few...

In the methods, please report the total number of animals used.

Abstract, line 1: Change “PSCs” to “PGCs”.

Significance statement, change “Our findings, we add...” to “Our findings add...”.

Page 8, top: “(below???)” looks like a leftover note from the authors.
